# Leaf as nursery: insights on hormonal control of asexual reproduction from *Kalanchoë pinnata*

**DOI:** 10.1093/jxb/eraf463

**Published:** 2025-12-04

**Authors:** Joanna Kacprzyk

**Affiliations:** School of Biology and Environmental Science, University College Dublin, Belfield, Dublin 4, Ireland

**Keywords:** Asexual reproduction, auxin, cytokinin, developmental plasticity, epiphyllous buds, gibberellic acid, inducible reproduction, Kalanchoë pinnata, phytohormones

## Abstract

This article comments on:

**Jácome-Blásquez F, Ooi JP, Viveros-Sánchez IM, Spencer V, Gündoğmuş YB, Kim M.** 2025. Plant hormones regulate inducible asexual reproduction in *Kalanchoë pinnata*. Journal of Experimental Botany **76**, 6896–6910. https://doi.org/10.1093/jxb/eraf405

This article comments on:

**Jácome-Blásquez F, Ooi JP, Viveros-Sánchez IM, Spencer V, Gündoğmuş YB, Kim M.** 2025. Plant hormones regulate inducible asexual reproduction in *Kalanchoë pinnata*. Journal of Experimental Botany **76**, 6896–6910. https://doi.org/10.1093/jxb/eraf405

**How does a leaf become a nursery for baby plants? In their study, Jácome-Blásquez *et al*. (2025) investigated *Kalanchoë pinnata*, a succulent that produces genetically identical plantlets along its leaf margins, to elucidate the hormonal signaling network that orchestrates this form of vegetative reproduction. Using exogenous hormone treatments, transgenic plants with altered hormone signaling, and live imaging of fluorescent reporter lines, the authors dissected the roles of auxin, cytokinin, and gibberellins in regulating the initiation and timing of plantlet formation. This work sets the foundation for a developmental model that has both evolutionary and practical relevance.**

Plants have a variety of asexual reproduction modes, including vegetative reproduction, somatic embryogenesis, and apomictic seeds (Schmidt *et al*., 2015). The *Kalanchoë* genus shows a unique asexual reproduction strategy, where plantlets are formed directly on the leaf margin (Garces *et al*., 2007). The reproduction through plantlet formation in *Kalanchoë* may have evolved as a trade-off with sexual reproduction (Jácome-Blásquez *et al*., 2022). Species that form plantlets constitutively, such as *K. daigremontiana*, have lost sexual reproduction; however, in species such as *K. pinnata*, the plantlet initiation is triggered by leaf detachment, and both sexual and asexual modes of reproduction co-exist (Garces *et al*., 2007). These inducible plantlets develop from secondary stem cell niches known as epiphyllous buds (EBs), which remain dormant until the parent leaf is detached (Naylor, 1932; Sawhney *et al*., 1994). The inducible plantlet formation involves co-opting of meristem and somatic embryogenesis pathways in the leaves (Jácome-Blásquez and Kim, 2023). *Kalanchoë pinnata* thus offers a window into conditional developmental reprogramming.

From a developmental biology standpoint, this system raises many interesting questions: What triggers meristematic reprogramming in somatic leaf cells? How is dormancy maintained and released? What signals can determine the spatial patterning of plantlet emergence? The research by Jácome-Blásquez *et al*. (2025) answered these questions in the context of plant hormone signaling (Fig. 1).

**Fig. 1.**
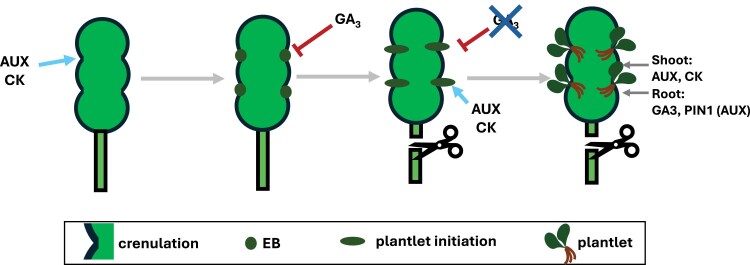
Auxin, cytokinin, and gibberellin as a hormonal triad controlling inducible plantlet formation on the leaf margins of *K. pinnata.* During early leaf development, auxin (AUX) and cytokinin (CK) drive the formation of crenulations. Crenulations are required for the subsequent development of epiphyllous buds (EBs). In attached mature leaves, the EBs are kept dormant by gibberellic acid (GA_3_). Once the leaf is detached, GA_3_ depletion and accumulation of AUX and CK in the EBs induce plantlet initiation. The interactive feedback network between the hormones regulates further plantlet development.

## Architects of plantlet formation: auxin and cytokinin

Auxin and cytokinin are classical plant hormones involved in nearly all aspects of growth and development, including meristem formation and maintenance (Davies, 2010). Their role appears no less central in *K. pinnata*. The authors investigated auxin (*DR5*::*GFP*) and cytokinin (*TCSn*::*GFP*) reporter lines during leaf development. The pattern of auxin and cytokinin activity suggested that they may act synergistically to establish the leaf crenulations: the small notches along the edge of a leaf that are the sites of plantlet formation. Auxin and cytokinin possibly also played a role in inducing expression of meristem genes required to form EBs (Jácome-Blásquez and Kim, 2023), as green fluorescent protein (GFP) signals were also visible in the centres of the EBs of mature leaves. After leaf detachment, there was a strong accumulation of GFP signals in plantlet primordia, suggesting that after EB dormancy is broken, auxin and cytokinin are involved in plantlet development. This model has been further validated by functional assays using antisense (AS) lines targeting the auxin transporter gene *KpPIN1* and the cytokinin signaling component *KpAHP*. Suppressing the expression of these genes led to abnormal leaf morphology, with both fewer crenulations and fewer plantlets. These findings are consistent with studies in Arabidopsis, where auxin transport through PIN proteins shapes the leaf serration pattern (Bilsborough *et al*., 2011) and cytokinin signaling regulates shoot apical meristem (SAM) formation and maintenance (Zhao, 2010; Chickarmane *et al*., 2012).

## The dormancy keeper: gibberellin

Auxin and cytokinin set the stage for plantlet development in *K. pinnata*, but the curtain appears to be controlled by gibberellic acid (GA_3_). Jácome-Blásquez *et al*. (2025) demonstrated that GA_3_ maintains EB dormancy in attached leaves, and that its depletion is necessary for plantlet initiation after leaf detachment. Exogenous GA_3_ application inhibited plantlet formation, whereas treatment with the GA_3_ antagonist paclobutrazol (PBZ) induced earlier and more abundant plantlet development. These results are consistent with previous observations that gibberellin restricts meristematic activity (Hay *et al*., 2002). The role of GA_3_ in maintaining dormancy was further supported by the phenotype of AS lines with reduced expression of a gene encoding a gibberellin-inactivating enzyme (KpGA2ox2), which displayed delayed or failed plantlet emergence that could be rescued by application of PBZ. This mirrors findings in Arabidopsis where seeds of *GA2ox2* loss-of-function mutants failed to germinate due to the accumulation of GA_4_ (Yamauchi *et al*., 2007). Taken together, the data supported a model in which GA_3_ acts as a suppressor of EB activation, and its inactivation via KpGA2ox2 is a key trigger for plantlet initiation upon leaf detachment.

## Hormonal networks during plantlet formation

*Kalanchoë pinnata* provided an elegant model to study the hormonal cross-regulation in a developmentally unique context. The authors demonstrated how the modulation of one hormone affected the others. For example, down-regulation of *KpPIN1* led to reduced expression of *KpYUC1*, an auxin biosynthesis gene, and *KpAHP* AS lines showed down-regulation of *KpPIN1* and *KpYUC1*. This suggests that auxin and cytokinin do not act in isolation and that there is a feedback loop in which transport and synthesis of these hormones are co-regulated. The down-regulation of *KpGA2ox2* in the AS lines also resulted in a slight down-regulation of *KpAHP* and *KpPIN1* genes, suggesting interactions between auxin, gibberellin, and cytokinin pathways during leaf development and plantlet formation in *K. pinnata.* These findings are in line with mechanisms previously described in Arabidopsis (Frigerio *et al*., 2006; Pernisova *et al*., 2016; Meng *et al*., 2017).

## Broader implications

The study advances the understanding of developmental plasticity in plants. The expression of meristem regulators such as *STM* and *CUC2* in *Kalanchoë* leaves (Garces *et al*., 2007; Jácome-Blásquez and Kim, 2023) indicates co-opting organogenesis and embryogenesis pathways during plantlet formation. The hormonal regulation demonstrated in the current study (Jácome-Blásquez *et al*., 2025) suggests the potential role of auxin, cytokinin, and gibberellin in the evolutionary repurposing of developmental programmes to establish a unique form of vegetative reproduction. Ecologically, inducible plantlet formation may offer survival strategies under volatile environmental conditions, with detached leaves acting as propagation units. Better understanding of the hormonal basis of this switch may assist conservation studies in species with similar reproductive strategies. In the future, especially once the downstream genetic networks responsible are characterized, lessons from *K. pinnata* have the potential to inform synthetic biology strategies for clonal propagation of other species. Inducing, controlling, or suppressing asexual reproduction could have a significant impact on agriculture and horticulture.

Collectively, *K. pinnata* emerges as a powerful model to study inducible asexual reproduction, offering valuable insights not only into developmental reprogramming but also into the potential manipulation of clonal propagation for both basic and applied plant science.
